# Calretinin-Periglomerular Interneurons in Mice Olfactory Bulb: Cells of Few Words

**DOI:** 10.3389/fncel.2016.00231

**Published:** 2016-10-07

**Authors:** Alex Fogli Iseppe, Angela Pignatelli, Ottorino Belluzzi

**Affiliations:** ^1^Biology and Evolution – Neurobiology, Department of Life Sciences and Biotechnology, University of FerraraFerrara, Italy; ^2^Department of Neurobiology, Physiology and Behavior, University of California at Davis, DavisCA, USA

**Keywords:** calretinin, olfactory bulb, periglomerular cell, patch clamp, signal-to-noise ratio

## Abstract

Within the olfactory bulb (OB), periglomerular (PG) cells consist of various types of interneurons, generally classified by their chemical properties such as neurotransmitter and calcium binding proteins. Calretinin (CR) characterizes morphologically and functionally the more numerous and one of the less known subpopulation of PG cells in the OB. Using of transgenic mice expressing eGFP under the CR promoter, we have tried to obtain the first functional characterization of these cells. Electrophysiological recordings were made in these cells using the patch-clamp technique in thin slices. Using ion substitution methods and specific blockers, we dissected the main voltage-dependent conductances present, obtaining a complete kinetic description for each of them. The more peculiar property of these cells from the electrophysiological point of view is the presence only of a single K-current, A-type – there is no trace of delayed rectifier or of Ca-dependent K-current. Other currents identified, isolated and fully characterized are a fast sodium current, a small L-type calcium current, and an inward rectifier, h-type cationic current. As a consequence of the peculiar complement of voltage-dependent conductances present in these cells, and in particular the absence of delayed-rectifier potassium currents, under the functional point of view these cells present two interesting properties. First, in response to prolonged depolarisations, after the inactivation of the A-current these cells behave as a purely ohmic elements, showing no outward rectification. Second, the CR cells studied can respond only with a single action potential to excitatory inputs; since they send inhibitory synapses to projection neurones, they seem to be designed to inhibit responses of the main neurones to isolated, random excitatory signals, rapidly losing their vetoing effect in response to more structured, repetitive excitatory signals. We propose that a possible role for these rather untalkative interneurons in the intense exchange of messages within the OB might be that of improving the signal-to-noise ratio in the first stages of the olfactory information processing.

## Introduction

Periglomerular (PG) cells of the olfactory bulb (OB) are interneurons which can be subdivided into distinct subtypes based on their neurochemical signature, generally determined by neurotransmitter and calcium binding proteins. The contribution of the different PG cells subtypes to signal processing within the bulbar network remains largely unidentified, and therefore knowing the functional properties of the single cell types may provide clues to fill this gap.

A relatively numerous group of calcium-binding proteins (CaBP) belonging to the EF-hand homolog family, capable to bind intracellular calcium with dissociation constants in the micromolar range (Dominguez et al., 2015) and including calretinin (CR), calbindin D-28k, neurocalcin, parvalbumin, and secretagocin, all characterize morphologically and functionally diverse subclasses of PG cells in the OB (Crespo et al., 1997; Alonso et al., 2001; Briñón et al., 2001; Kosaka and Kosaka, 2007, 2013; Parrish-Aungst et al., 2007).

Differential expression of CaBPs in PG cells is likely to translate in differences in the behavior of these cells, which would be responsible of the diversity in the modulation exerted on the projection neurons. The distribution and morphologic characteristics of CR neurones, object of this study, has been the target of several studies in the OB, accomplished using immunocytochemistry, radioimmunoassay, and *in situ* hybridization methods (Jacobowitz and Winsky, 1991; Résibois and Rogers, 1992; Bastianelli and Pochet, 1995; Kosaka and Kosaka, 2012; Krosnowski et al., 2012). However, as for other PG cells subpopulations, the very accurate morphological descriptions available (Kosaka and Kosaka, 2007, 2009; Panzanelli et al., 2007; Batista-Brito et al., 2008; Krosnowski et al., 2012), is not paralleled by an equally accurate knowledge of their functional properties. This is mainly due to the difficulty in recognizing the different PG cell subtypes in living preparations. The use of transgenic mice is progressively filling this gap for many CaBP-containing neurons in various areas of the CNS (Schwaller, 2012), but so far not for the olfactory bulb.

There are several reasons to investigate the contribution of these cells in the bulbar circuitry. First they are abundant in the glomerular layer, being the more numerous among the cells immunoreactive for CaBP (Crespo et al., 1997) and -as for the other PG cells- our knowledge of their contribution to the processing of the incoming sensory information is fragmentary and largely incomplete. In the useful classification proposed by Kosaka, these cells pertain to type-2 PG cells, i.e., cells that do not receive direct input from olfactory nerve terminals (Kosaka et al., 1997), but establish direct inhibitory synapses onto the apical dendrites of projection neurones (mitral and tufted cells) (Batista-Brito et al., 2008), and therefore an influence in signal processing of some importance has to be expected from cells so strategically placed at the entry of the bulbar circuitry. Knowing the functional properties of the single cell types may provide clues to fill this gap.

A further reason of interest for this sub-population of PG cells is that CR-positive interneurons undergo adult neurogenesis (Kato et al., 1999; De Marchis et al., 2007; Batista-Brito et al., 2008; Liu and Guthrie, 2011).

In this work, based on a thorough analysis of their electrophysiological properties, we propose a possible role of a population of CR interneurons in the glomerular layer of mouse OB, suggesting that they could improve the signal-to-noise ratio in the first stages of the olfactory information processing.

## Materials and Methods

### Animals

#### Animal Ethics

A total of 97 mice of both sexes have been used; the average age was 45 ± 3.9 days (mean ± SE). Experimental procedures were designed so as to reduce the number of animals used and their sufferance. Care and use of animals was conducted according to guidelines established by European Council (63/2010) and Italian laws (D.Lgsl 26/2014) on the protection of animals used for scientific purposes. The experimental protocols were ratified by the Committee for Animal Welfare of the University of Ferrara (OBA), by the Directorate-General for Animal Health of the Ministry of Health, and were supervised by the Campus Veterinarian of the University of Ferrara.

#### CB2-eGFP Mice

Calb2 or CR-eGFP mice, strain Tg(Calb2-EGFP)CM104Gsat/Mmmh, were obtained from the Mutant Mouse Regional Resource Center (MMRRC); detailed information can be found at: http://www.mmrrc.org/strains/283/0283.html. Mice were generated on Swiss-webster background; transgenes (BAC) containing eGFP inserted upstream of targeted gene were injected into pronuclei of FVB/N fertilized oocytes; hemizygous progeny was mated to Swiss Webster mice each generation thereafter for reproduction (Gong et al., 2003).

CB2-GFP expression in the OB reflected the *in situ* distribution of mRNA for CR but, as already observed by other using the same transgenic mice, in the brain in general (Huberman et al., 2008) and in the OB in particular (personal observation, data not shown), there was less GFP than would be expected on the basis of CR mRNA expression (Gong et al., 2003) or on the basis of immunohistochemical studies (Panzanelli et al., 2007; Batista-Brito et al., 2008; Kosaka and Kosaka, 2009; Krosnowski et al., 2012), a discrepancy that could be accounted for positional effects of the CB2-GFP transgene insertion.

### Solutions

According to the objective of the experiment, different solutions have been used; all the concentrations listed below are expressed in mM.

For the dissection and slice preparation the solution used was:

• *High sucrose ACSF solution*: 215 sucrose, 3 KCl, 21 NaHCO_3_, 1.25 NaH_2_PO_4_, 1.6 CaCl_2_, 2 MgCl_2_, and 10 glucose.

During the electrophysiological experiments, several extracellular solutions (EC) have been employed to characterize the different conductances under study.

• EC1, *standard ACSF extracellular solution*: 125 NaCl, 2.5 KCl, 26 NaHCO_3_, 1.25 NaH_2_PO_4_, 2 CaCl_2_, 1 MgCl_2_, and 15 glucose.
• EC2, *high K^+^ plus TEA EC solution*: 70 NaCl, 32.5 KCl, 26 NaHCO3, 1.25 NaH2PO4, 2 CaCl2, 1 MgCl2, 20 TEA, and glucose.
• EC3, *no Na^+^ plus TEA EC solution*: 110 CholineCl, 2.5 KCl, 26 NaHCO3, 1.25 NaH_2_PO4, 2 CaCl2, 1 MgCl_2_, 20 TEA, and 10 glucose.

The osmolarity of all external solutions was adjusted at 305 mOsm with glucose. EC solutions are continuously bubbled with 95% O_2_ and 5% CO_2_ during the experiment. In all the recordings, except where indicated, kynurenic acid (1 mM) and bicuculline (10 μM) have been added to the EC to abolish glutamatergic and GABAergic synaptic activity.

The pipette-filling intracellular solution used contained (in mM):

• IC1, *standard solution*: 120 K-gluconate, 10 NaCl, 2 MgCl_2_, 0.5 CaCl_2_, 5 EGTA (ethylene glycol tetraacetic acid), 10 HEPES, 2 Na-ATP, and 10 glucose.
• IC2, *Cs solution*: 113 CsCl, 20 TEA, 10 NaCl, 2 MgCl_2_, 0.5 CaCl_2_, 5 EGTA (ethylene glycol tetraacetic acid), 10 HEPES, 2 Na-ATP, and 10 glucose.

The osmolarity of IC solution was adjusted to 295 mOsm with glucose, and the pH to 7.2 with KOH. The free calcium concentration with this internal solution was calculated to be 16 nM.

### Recording Conditions

Slices (200 μm) in the coronal plane were obtained using a Campden 752 vibroslice tissue cutter (Campden Ltd., Loughborough, England). Current and voltage recordings were acquired with an Multi-Clamp 700B amplifier (Molecular Devices, Sunnyvale, CA, USA), and a 12 bit A/D–D/A converter (Digidata 1440A; Molecular Devices). Borosilicate glass pipettes (1.5 O.D., 0.87 I.D., with filament; Hilgenberg, Malsfeld, Germany) were pulled with a Sutter P-97 puller (Novato, CA, USA) and had a resistance of 4–5 MΩ when filled with standard intracellular (IC) solution; the seal formation was realized with the help of an air pressure controller (MPI, Lorenz Messgerätebau, Katlenburg-Lindau, Germany); the seal resistance was always greater than 2 GΩ. The liquid-junction potential (LJP) of the different solutions was estimated using the junction potential calculator of pClamp (Molecular Devices).

### Data Analysis

Oﬄine analysis was performed using version 10 of pClamp (Molecular Devices), version 8 of Origin (OriginLab Corporation, Northampton, MA, USA) and Prism 5 (GraphPad). Unless otherwise indicated, data are presented as means ± SEM. Statistical significance of the results was calculated with Student’s *t*-test, one-way or two-way analysis of variance (ANOVA), as indicated. D’Agostino and Pearson omnibus test was used to check for normality. A *P*-value of <0.05 is considered significant.

A numerical model of CR PG cells has been developed according to the HH model (Hodgkin and Huxley, 1952). The program is written in Q-Basic and the code is available upon request.

## Results

### General Properties

The data are based on recordings from 280 CB2-eGFP (CR+) PG neurons from the glomerular layer. The CR+ cells are smaller than other types of PG interneurons, with a membrane capacitance of 4.07 ± 0.24 pF (*n* = 280) and an input resistance (1877.7 ± 114.3 MΩ; *n* = 270); the diameter was 6.44 ± 1.83 μm (*n* = 20), in close agreement with what has been reported in another study (Kosaka and Kosaka, 2007) for a much larger group of cells (6.66 ± 0.12, *n* = 338).

**FIGURE 1:**
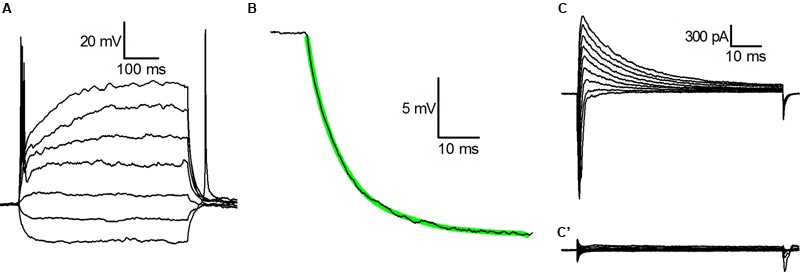
**Basic properties of CR-GFP cells. (A)** Whole cell recordings in current-clamp mode; voltage responses to injected currents (from -15 pA, in increments of 10 pA) from a membrane potential of -70 mV. (B) Current-clamp voltage transient in response to the injection of -15 pA; the thick green line is the best least squares fit with a single exponential of the experimental recording. (C) Whole cell recordings (cell in voltage-clamp mode; family of currents evoked by voltage steps to potentials ranging from -40 to +40 mV after 250 ms preconditioning at -100 mV. (C’) Same as (C), but after a preconditioning at -50 mV.-

**Figure 1A** shows the voltage response of a typical CB2-GFP PG cell to injected currents from a holding potential of -70 mV; in 82% of the cells analyzed for this aspect (*n* = 61) we observed a single action potential in response to the injection of depolarising current steps, and do no sign of outward rectification, compelling the cells to behave as ohmic elements as normally observed only at hyperpolarized potentials; the remaining 18% of the cells were unable to generate action potentials and showed no outward rectification.

The voltage trajectories in response to hyperpolarizing pulses were further analyzed with the method of the least-squares fit to determine the electrotonic compactness of the structure: in nearly all the cells studied the trajectories accurately followed a single exponential (**Figure 1B**), a behavior usually taken as an indication that the soma of the neurone under study behaves as a single, nearly isopotential compartment (Brown et al., 1981).

The main Na^+^ and K^+^ voltage-dependent currents that can be activated in these cells under voltage-clamp conditions, are shown in **Figure 1C**: depolarizations to potentials ranging from -40 to +40 mV after 250 ms preconditioning at -100 mV, besides the fast transient Na^+^ current could evoke only a single outward current, a large, A-type K^+^ current, whereas the delayed rectifier potassium current is virtually absent. Indeed, depolarizations to the same voltages from a holding potential of -50 mV do not evoke any current. As a consequence, following sustained depolarizations leading to inactivation of *g*_Na_ and of *g*_A_ (**Figure 1C**’), these neurons behave as purely ohmic elements, as it can be observed in **Figure 1A**.

The cells correspond to what, in a previous paper (Puopolo and Belluzzi, 1998), has been described as N (non-rectifying) PG cells.

### *I*_A_

**FIGURE 2:**
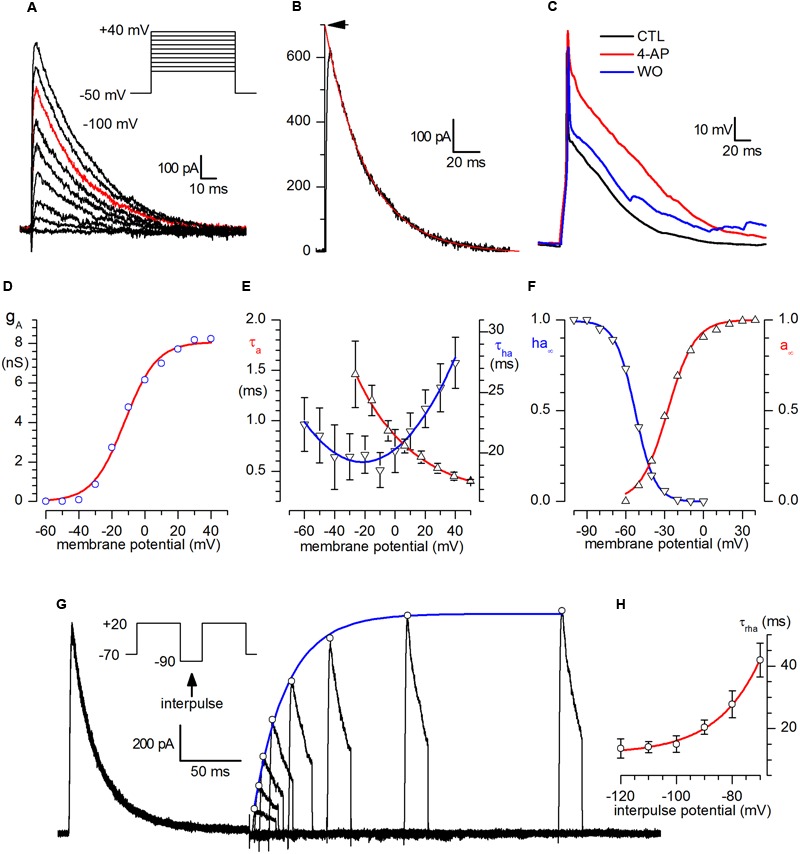
**A-current. (A)** Family of A-currents recorded following the protocol shown in the inset after *I*_Na_ block with 1.2 μM TTX and suppression of spontaneous synaptic currents with kynurenate (1 mM) and bicuculline (10 μM). **(B)** Example of analysis of the decaying phase of the current. The inactivation time constant, τ_a_, was computed from the least-squares fit of the current decay, taken from twice the time-to-peak to the end of the depolarizing pulse. The extrapolation of the exponential to time zero (arrow) gives the open-channel current at this voltage. The trace shown (represented in red in the family shown in A) was obtained by a depolarization to +20 mV after complete removal of inactivation. **(C)** Analysis of the contribution of the A-current to the repolarizing phase of the action potential: current-clamp recordings from a holding potential of -70 mV in normal saline (black), in the presence of 3 mM 4-AP (red) and after washout (blue) – experiment replicated in nine cells. **(D)** Voltage dependence of A-conductance. *g_A_(V)* was calculated from *I_A_(V)/(V - E_K_*), using *I_A_(V)* values corrected for inactivation, obtained by extrapolating the current decay at the zero time, and *E*_K_ = -101 mV. The interpolating line is the fit with equation 1 (see text), with *V*_h_ = -12.3 mV, *z* = +3.8 and *g*_A.max_ = 8.08 nS. **(E)** Voltage dependence of activation (τ_A_, upward triangles) and inactivation (τ_ha_, downward triangles) time constants. τ_A_, calculated from the time-to-peak, as explained in the text for the sodium current, in the voltage range analyzed could be described phenomenologically by the equation τ_A_(*V*) = 0.52^∗^exp(-*V*/34.6) + 0.26; in the same voltage range, τ_ha_ dependence voltage (downward triangles) could be phenomenologically described by the equation τ_ha_ = 20.32+0.09846^∗^*V*+0.00227^∗^*V*^2^. **(F)** Steady-state activation (a_∞_, upward triangles) and inactivation (ha_∞_, downward triangles) curves for the A-channel. The continuous line, resulting from the analysis of a 12-neuron sample, obeys the equation h_∞_(*V*) = 1/[1+exp(*V* -*V*_50_)/*k*_ha_)]. **(G)** *I*_A_ removal of inactivation. Double-pulse protocols (inset) produced transient outward currents of increasing amplitude following increments of the interpulse width. The peaks of the outward current (open circles) are interpolated with an exponential function. The fitted curve (single exponential) gives a time constant (τ_ha_ = 23.5 ms) for the removal of inactivation in the cell shown for that particular de inactivating potential (-90 mV). **(H)** Voltage dependence of the time constant of removal of inactivation, τ_rha_ between -120 and -70 mV; the interpolating line, obeying the equation τ_rha_ = 2968.8 ^∗^ exp(-*V*/-15.27) + 11.86, provides a continuous phenomenological description of τ_rha_ in the range analyzed.

After *I*_Na_ block with 1.2 μM TTX, *I*_Ca_ block with Cd^2+^ 100 μM and suppression of spontaneous synaptic currents with kynurenate (1 mM) and bicuculline (10 μM), a virtually pure I_A_ remained in CR-GFP PG cells, as shown in **Figure 2A**. The current was blocked by 3 mM 4AP (*n* = 11; not shown); the contribution of the current in the repolarizing phase of the action potential is illustrated in **Figure 2C**.

The A-current activation process is illustrated in **Figures 2 A,B,D–F**. The current develops following a third-order exponential (not shown). The activation time constant, τ_a_, studied in a 6-neuron sample in the -30 to +40 mV range, was computed from the least-squares fit of a cubic exponential to the rising phase of the A-current; the upward triangles in **Figure 2E** (left scale) describe its dependence upon voltage in the range studied.

The open channel current as a function of voltage, was obtained from the extrapolation at zero time of the decaying phase of the current, (point marked by an arrow in **Figure 2B**) in a 20-neuron sample (not shown). From this relation, the open channel conductance-voltage relationship, *g*_A_(*V*), was then computed by dividing the current at the zero time by the driving force at the different potentials. In the assumption that the channels obey a Boltzmann distribution, in which the ratio of open to closed channels is an exponential function of voltage *V*, being low at negative voltage and approaching infinity for large positive voltages, then the sigmoidal *g*_A_(*V*) shown in **Figure 2D** can be described by the equation (Ehrenstein et al., 1970):

(1) $$g_{\text{A}}(V)=g_{\text{A},\max}{\left[1+\exp\frac{ze}{kT}(V_{0}-V)\right]}^{-1}$$

where *V*_0_ is the voltage expressing the half-maximal conductance *g*_A,max_, *z* the valence of the single voltage-sensing particle, *e* the electronic charge, *k* the Boltzmann constant, and *T* the absolute temperature. The continuous curve in **Figure 2D** is a plot of equation (1) with *g*_A,max_ = 8.1 nS, *z* = +3.8 and *V*_0_ = -12.3 mV.

The voltage dependence of the steady-state activation parameter, a_∞_, was computed by extracting the cubic root from the ratio *g*_A_(*V*)*/g*_A,max_ (**Figure 2F**, red line). *a*_∝_(V) had a midpoint (*V*_50_) at -27.6 mV and a slope, *k*_a_, of 10.4 mV.

The voltage-dependence of the steady-state inactivation curve is illustrated in **Figure 2F** for a 12-neuron sample. The inactivation curve, computed independently for each cell, had a midpoint centered at -52.7 ± 0.36 mV and a slope, *k*_ha_ of 7.59 ± 0.32 mV (**Figure 2E**, blue line).

The inactivation time constant, τ_ha_, was computed by interpolating a single exponential to the current decay (**Figure 2B**), and showed little voltage dependence at the potentials considered (**Figure 2E**, blue line).

The activation time constant was calculated numerically from time-to-peak of the current and inactivation time constant, assuming an activation kinetic of four (Bonifazzi et al., 1988) and resolving the equation 2 of the paper with the Newton’s method (Süli and Mayers, 2003); its dependence on voltage is represented in **Figure 2E**, and the relative equation describing phenomenologically the process is indicated in the figure legend.

Finally, removal of inactivation was studied with double-pulse protocols (**Figure 2G**) and its time constant, τ_rha_, ranged from 41.9 ms at -70 mV to 13.6 ms at -120 mV (*n* = 4, **Figure 2H**).

### *I*_Na_

**FIGURE 3:**
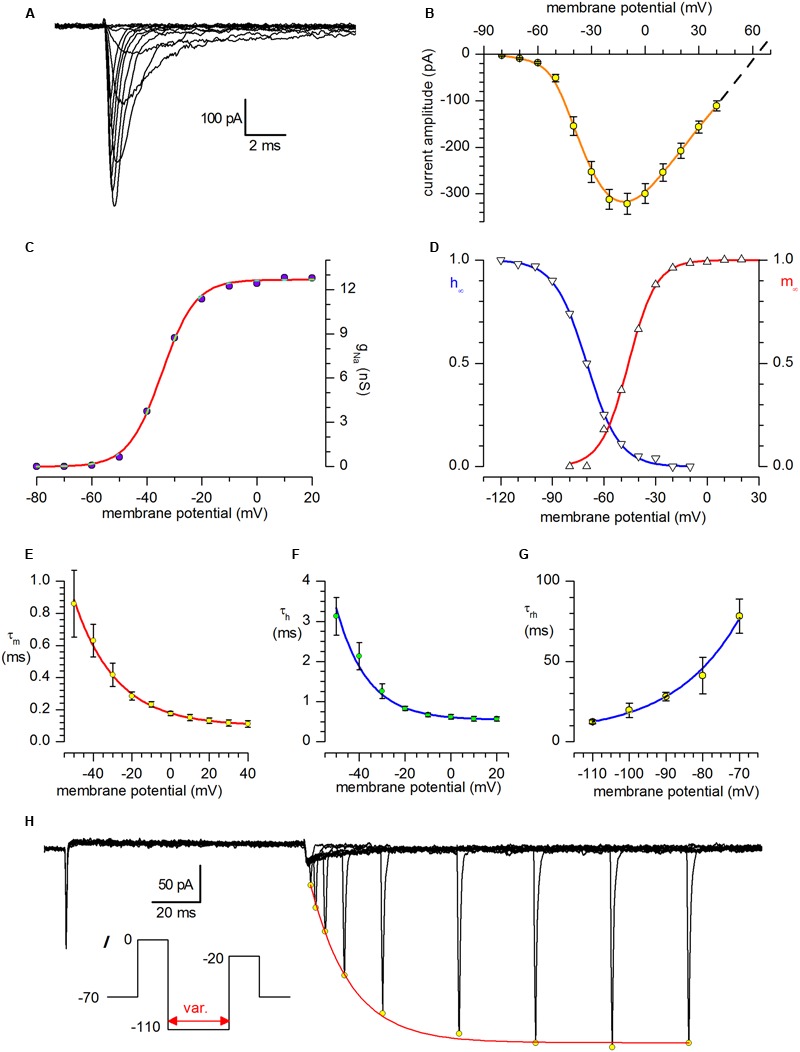
**Sodium current. (A)** Family of sodium currents recorded after *I*_K_ block with and suppression of spontaneous synaptic currents with kynurenate (1 mM) and bicuculline (10 μM). The holding potential was **-**70 mV, and the depolarising pulses, in 10 mV increments, were preceded by 250 ms preconditioning at **-**100 mV; internal solution IC2 plus extracellular Cd^2+^ 100 μM. **(B) I–**V relationship for *I*_Na_; mean amplitudes ±SE from 31 different neurones. The dashed line is the linear regression of the last four values extrapolated to the abscissa to calculate the reversal potential; the theoretical *E*_Na_ is +61.4 mV. **(C)** Voltage dependence of Na conductance. *g*_Na_(*V*) was calculated from *I*_Na_(*V*)/(*V* **-** *E*_Na_), using *I*_Na_(*V*) values given corrected for inactivation obtained by extrapolating the current decay at the zero time, and *E*_Na_ = +63 mV. The interpolating line is the fit with the Boltzmann equation, *g*_Na_(*V*) = *g*_Na,max_/(1 + exp((*V*_h_ **–** *V*)*ze*/*kT*)**^-^**^1^), with *V*_h_ = **-**34.6 mV, *z* = +4.3, and *g*_Na.max_ = 12.7 nS. **(D)** Steady-state activation (m_∞_) and inactivation (h_∞_) curves for the Na-channel. The continuous lines, resulting from the analysis of a 31-neuron sample for activation and 23 neurones for inactivation, obeys the Boltzmann equation h_∞_(*V*) = 1/**[**1 + exp((*V* **-** *V*_50_)/*k*_ha_)**]** with midpoints of **-**45.8 and **-**70.0 mV and slopes of 8.1 and 9.4 for activation and inactivation, respectively. **(E)** Activation time constant, τ_m_, at potentials from **-**110 to **-**70 mV; the continuous line, a purely phenomenological description of τ_m_ vs. voltage, obeys the equation τ_m_ = 0.07796 * exp (**-***V*/21.752) + 0.00992. **(F)** Inactivation time constant, τ_h_, at potentials from **-**50 to +20 mV; the continuous line is a phenomenological description of τ_h_ vs. voltage, and obeys the equation τ_h_ = 0.0679 * exp (**-***V*/13.5) + 5.43. **(G)** Time constant for removal of inactivation, τ_rh_, at potentials from **-**110 to **-**70 mV; the continuous line, a purely phenomenological description of τ_rh_ vs. voltage, obeys the equation τ_rh_ = 3853 * exp(**-***V*/**-**17.58) + 5.11. **(H)** *I*_Na_ removal of inactivation. Double-pulse protocols (inset) produced transient outward currents of increasing amplitude following increments of the interpulse width. The peaks of the outward current (open circles) are interpolated with an exponential function. The fitted curve (single exponential) gives a time constant for the removal of inactivation, τ_ha_, of 16.8 ms in the cell shown.

The sodium current was studied using Cs saline as internal solution and Cd 100 μM in external solution. A well-developed *I*_Na_ was present in all the cells studied; a family of sodium currents activated in the voltage range from -80 to +40 mV can be seen in **Figure 3A**.

The I–V relationship registered a maximum at about -10 mV, and is shown in **Figure 3B** for a 31 neurone sample; the dashed line has been calculated as linear regression of the last four values and extrapolated to the *x*-axis to calculate the reversal potential; for comparison, the nernstian E_Na_ is +61.4 mV. The conductance-voltage relationship, *g*_Na_(*V*), computed by dividing the extrapolation at the zero time of the decaying phase of the current by the driving force, as explained above for the A-current, was described by the Boltzmann equation with z = +4.3 and a maximal conductance of 12.7 nS (**Figure 3C**).

The steady-state activation and inactivation curves are shown in **Figure 3D**, and the parameters of the Boltzmann curves describing the two processes are indicated in the figure legend.

The activation and inactivation time constants (τ_m_ and τ_h_, **Figures 3E,F**) is relatively fast; of some interest is the analysis of the removal of inactivation (**Figures 3G,H**), which shows unusually high time constants (about 80 ms at -70 mV), which, combined with the faster removal of inactivation of the A-current (40 ms at the same potential), can explain the poor capacity of these cells to fire repetitive action potentials (see *Numerical Model* and *Discussion* below).

### *I*_h_

**FIGURE 4:**
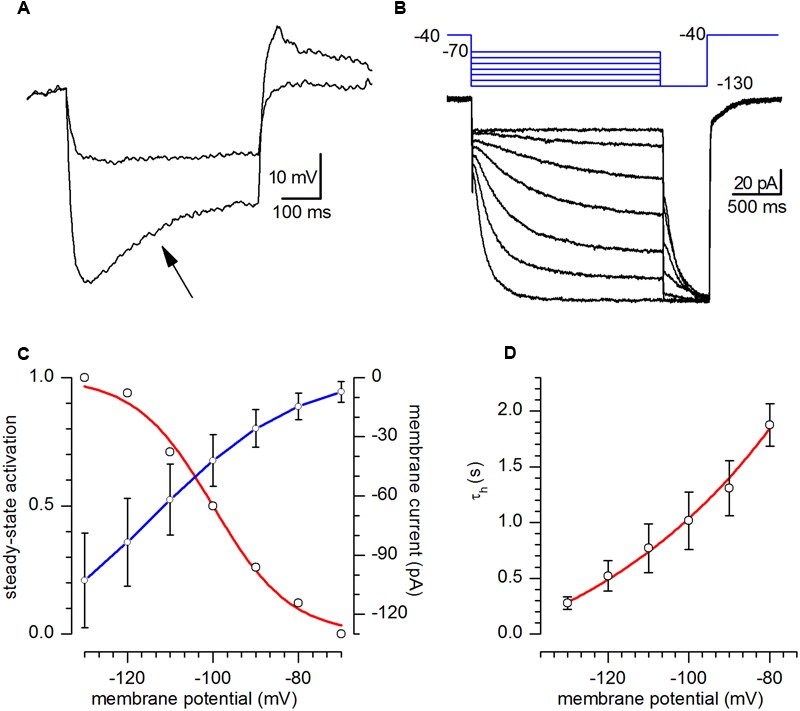
**h-current. (A)** Response of a CR-GFP neuron under current-clamp condition to the injection of 5 and 15 pA hyperpolarizing current pulses. Note the appearance of the archetypal sag (arrow) due to the activation of *I*_h_; bath solution for this recording was standard ACSF. **(B)** Family of responses of a CR-GFP neuron under voltage-clamp conditions to the application of the double pulse protocol shown in the inset above; explanation in the text; bath solution EC2. **(C)** Current–voltage relationship of the h-current (blue line, right y-axis) and voltage-dependence of the activation curve (red line, left y-axis) obtained from tail analysis of double-pulse experiments as shown in **(B)**; mean value ± SE; (*n* = 20). **(D)**. *I*_h_ activation time constant as a function of membrane potential; the continuous line, phenomenological description of τ_h_ in the voltage interval shown, obeys the equation τ_h_(*V*) = 5908 ^∗^ exp[-*V*/(-51.82) - 677.48].

The h-current was studied by using extracellular solution EC2, designed to enhance its amplitude. Under current-clamp conditions all of the examined CR neurons showed the typical time-dependent depolarizing sag in response to the injection of hyperpolarizing currents suggestive of the presence of a h-current (**Figure 4A**, arrow); in all the cells studied the depolarizing sag became evident at potentials more negative than -90/-100 mV, increased in amplitude at more negative potentials and at the termination of the hyperpolarising current pulse was usually followed by a depolarising overshoot.

In voltage-clamp conditions, hyperpolarizing commands from a holding potential of -40 mV evoked slow inward relaxations over the same membrane potential range as the ones producing sags and depolarizing overshoots in hyperpolarizing potentials (**Figure 4B**). The h-current activated slowly and increased its amplitude and rate of activation as the cells were progressively hyperpolarized, with no sign of inactivation. Two components of the current were measured during the hyperpolarizing voltage steps: (i) an instantaneous current (*I*_inst_), obtained at the beginning of the step, and (ii) a steady-state current (*I*_ss_), obtained at the end of the step. The instantaneous current was almost linear along the explored voltages, while the steady-state current increased its magnitude as the membrane potential was made more negative; the h-current amplitude, measured as *I*_ss_–*I*_inst_ is plotted against voltage in **Figure 4C** (blue). The steady-state activation curve (**Figure 4C**, red) was obtained by interpolating the relative amplitudes of the tail currents with the Boltzmann function, finding values for half-activation (V_50_) of -100 ± 0.82 mV and of 9.02 ± 0.74 mV for the slope (*n* = 6).

The h-current time course could be fitted by a single exponential, whose time constant, as a function of voltage is represented in **Figure 4D**.

### *I*_Ca_

A small persistent inward current was detected during prolonged depolarizing pulses after blockage of sodium and potassium channels which, for its biophysical and pharmacological properties, has been identified as a L-type calcium current [*I*_Ca(L)_].

**FIGURE 5:**
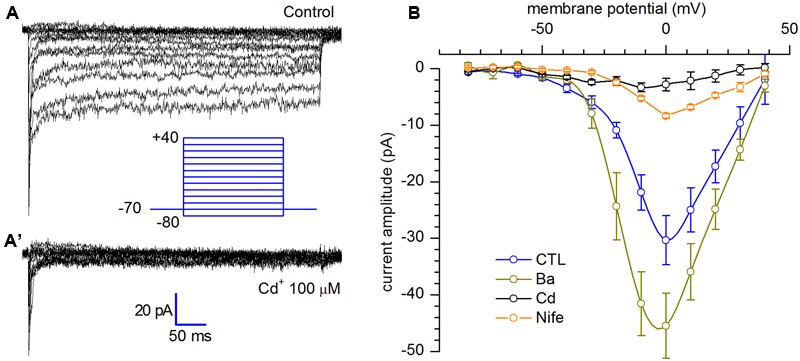
**Calcium current (A)** L-type calcium current activation. Persistent calcium currents recorded in response to depolarizing voltage steps ranging from -80 to +40 mV (10 mV increments), from a holding potential of -70 mV; **(A′)**. After application of Cd^2+^ 100 μM; tracings recorded using EC3 bathing solution. **(B)** I–V relationships in control (*n* = 13), after substitution of Ca^2+^ with 4 mM Ba^2+^ (*n* = 12), 20 μM nifedipine (*n* = 11) and 100 μM Cd^2+^ (*n* = 3); current amplitudes were measured averaging the last 10 ms at the end f each step.

The voltage clamp experiments shown in **Figure 5** have been realized using the extracellular solution EC3, in which sodium was substituted for by an equimolar amount of cholineCl in order to suppress the *I*_Na_, and intracellular solution IC2, in which potassium was substituted by an equimolar amount of caesium (further details in Solutions). In addition, the block of the I_A_ has been achieved with the simultaneous addition of 20 mM TEA and replacement of K^+^ with Cs^+^ in the intracellular solution. The protocol applied to evoke the current presented voltage commands ranging from -80 to +40 mV, starting from a holding potential of -70 mV (**Figure 5A**). The current, showing a maximal open channel conductance of about 0.6 nS with Ca^2+^, was potentiated by 4 mM Ba^2+^ and inhibited by 20 μM nifedipine and 100 μM Cd^2+^ (**Figure 5B**).

### Functional Properties

In these neurones, the excitability profile is determined by two transient currents, *I*_Na_ and *I*_A_, both subject to complete inactivation. A remarkable kinetic property of the fast transient sodium current in these cells is its relatively long time constant for removal of inactivation (about 80 ms at -70 mV, **Figure 3G**), implying that once the conductance has entered into the inactivated state, the membrane has to return to negative potentials for several tens of milliseconds before a sufficient number of Na-channels become re-excitable. It is of note that the only potassium current displayed by these cells is a large, A-type fast transient outward current, whereas the delayed rectifier potassium currents, including the Ca-dependent, are virtually absent. The result of this unusual complement of voltage-dependent conductances, is that sustained depolarizations leading to inactivation of sodium and A-type conductance, change these neurons into, purely ohmic (**Figure 1C**’), unexcitable elements, as it is possible to observe in the almost linear I/V relationship of the late phase of the current-clamp tracings shown in **Figure 1A**.

**FIGURE 6:**
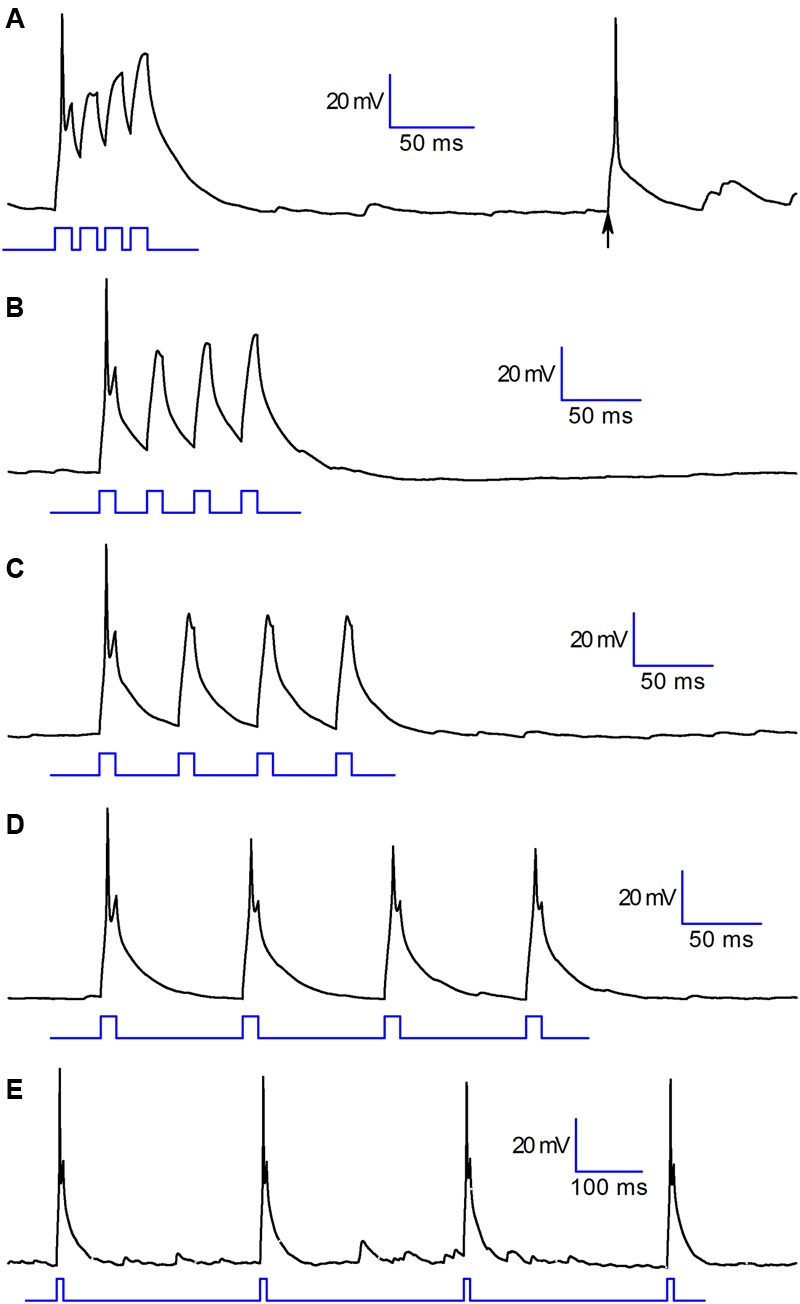
**Functional properties.** Voltage response of a CR+ PG cell to 10 ms depolarizing pulses at different time intervals: (in ms) 15 **(A)**, 30 **(B)**, 50 **(C)**, 90 **(D)**, 310 **(E)**. Note the full-size action potential marked by an arrow in trace A, resulting from random excitatory input. This behavior has been observed without exception in six cells.

Under the functional point of view, the consequence of this combined provisions is illustrated in **Figure 6** (experiment replicated in six cells): the cell readily responds to a depolarising step with a single action potential, but then becomes unexcitable for several tens of milliseconds – the permanence for at least one hundred milliseconds at the resting potential is necessary to allow the cell to return to an almost normally responsive state, reacquiring the capability to generate action potentials (**Figure 6**). If the cell receives an excitatory input it will respond by generating a single action potential (A,B,C) that will inhibit the projection neurons. Spare excitatory inputs (arrow in A), as those due to random noise, would translate into an activation of these cells, and consequently into an inhibition of projection neurons. More complex is the response to trains of close excitatory inputs, as those due to repetitive discharge from the olfactory nerve following the activation of olfactory sensory neurons. In this case, the first excitatory input would still lead to a transient inhibition of the projection neurons, as seen above for random (noise) signals, but the subsequent inputs will not be capable to evoke action potentials in CR interneurons, which would then end their inhibitory action on projection neurons. This behavior has been observed without exception in six cells.

### Numerical Model

**FIGURE 7:**
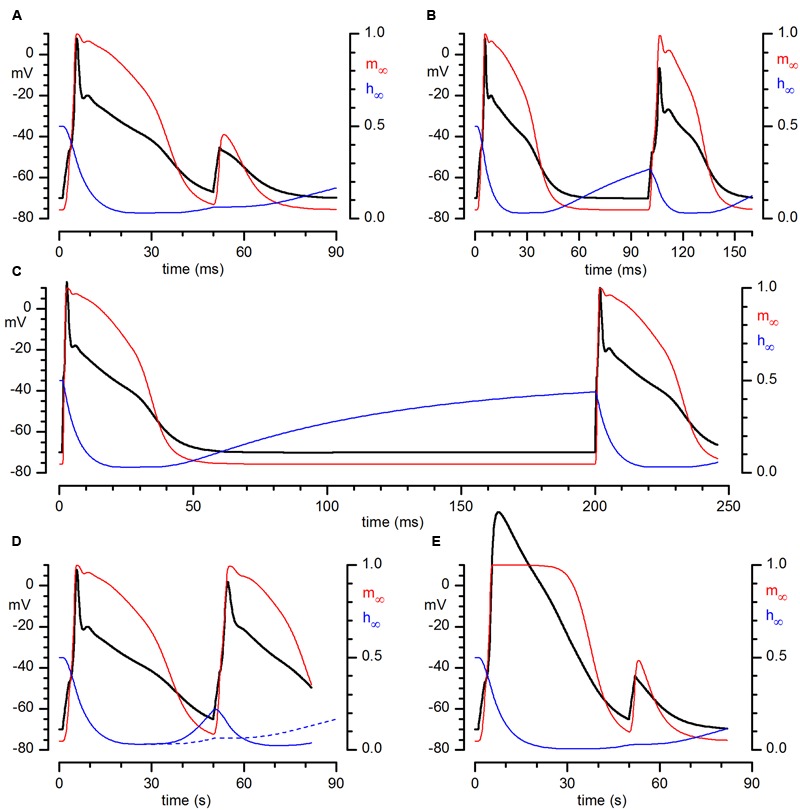
**Numerical reconstruction of the action potential in CR+ PG cell.** Numerical reconstruction of voltage responses of a CR+ PG cell to depolarizing train pulses at different time intervals: (in ms) 50 **(A)**, 100 **(B)**, and 200 **(C)**. In color are represented the probabilities of single activation (red) and inactivation (blue) gates of the sodium current to be in the open state during the development of the action potentials. **(A–C)** Result of the simulation of injection of just-above threshold depolarizing pulses at 50, 100, and 200 ms intervals. **(D)** Same experiment as **(A)** (50 ms interval), but after a reduction of the time constant for removal of inactivation by a factor 10. The dashed blue line is the trajectory of the inactivation gate of the simulation in **(A)**, for comparison. **(E)** Same simulation conditions as in **(A)**, but in this case after suppression of the A-conductance.

The analysis of the electrical properties of bulbar CR neurones has been completed by a numerical model of these cells in Hodgkin-Huxley terms (Hodgkin and Huxley, 1952), considering the cell as a single electrical and spatial compartment. The model incorporates Na, A and Ca conductances. All the equations and parameters used, as well as the assumptions made, are listed in the Supplementary material. The solution of the set of differential equations describing the kinetics of the currents considered produced the tracings shown in **Figure 7**. The model is capable of capturing fairly well the essential features of the excitability profile of these cells, producing full-size action potentials which show the same development in time as recorded spikes and providing the same responses to multiple stimulations at different intervals shown in **Figure 6**. In addition to the voltage trajectories, in **Figure 7** are illustrated also the behaviors of activation (red) and inactivation (blue) gates during the process. **Figures 7A–C** show the result of the simulation of injection of depolarizing pulses at 50, 100, and 200 ms intervals, respectively – note how the inactivation gate after 100 ms is only partially de-inactivated, barely supporting the Hodgkin cycle (B), and how after 200 ms (C) it is almost fully de-inactivated, returning to the steady-state value.

The weight of the long time for removal of inactivation of the sodium channel in determining the repetitive firing capability of these neurones is shown in the simulation illustrated in **Figure 7D**: in this case we have replicated the same simulation illustrated in panel A (two just-above threshold stimuli separated by 50 ms) but shortening the time constant for removal of inactivation by a factor 10. Note that now an almost full-size action potential develops.

The contribution of the A-current to the firing of CR PG cells is illustrated in **Figure 7E**, a replica of the simulation of **Figure 7A** in which the A-conductance has been suppressed – compare the result of this simulation with **Figure 2C**, showing the result of the blockage with 4AP.

The model allows an effective evaluation of the accuracy of the kinetics calculated, and an in-depth understanding of the details of the mechanisms responsible for the inability of these cells to fire multiple action potentials, confirming that this depends on the long time required to remove the inactivation of the sodium channel.

## Discussion

This study provides a description of the functional properties of a population of CR+ PG cells in mouse OB, providing baseline information about the functional properties of CR-containing interneurons that can be useful in further studies aiming at better understanding the signal processing taking place in the olfactory system. The main result is that these cells present a set of voltage-dependent currents dominated by two fast transient currents, sodium and potassium A-type, making them capable to respond only with a single action potential to excitatory stimuli, whether isolated or repetitive, before entering into an inactivated state.

The peculiar complement of voltage-dependent conductances present in these cells is evocative of the excitability profile characterizing newborn PG neurons (Belluzzi et al., 2003), and it has been demonstrated that CR PG cells are generated also in adulthood (Kato et al., 1999; De Marchis et al., 2007; Batista-Brito et al., 2008; Liu and Guthrie, 2011). However, the possibility that the CR+ cells studied were a fraction of newly generated neurones can be excluded indirectly by the observation that all the cells studied were a highly homogeneous population showing the same electrophysiological profile and, more directly, by experimental evidence recently provided by a group working in a very similar transgenic mice model using BrdU birth dating experiments, that CR-GFP PG cells maintain their typical excitability profile based on A- and Na currents “for weeks if not months” after BrdU injection (Desaintjean et al., 2016).

### CR Neurones Connectivity in the OB

The synaptic connections of the different PG cells in the glomerular layer is clearly a fundamental question when trying to understand their role, but regrettably our knowledge of the synaptic connectivity in the glomerular neuropile is scarce, under several aspects, rather inconsistent (Vaaga and Westbrook, 2016). The CR PG cells are no exception, but there are few clues which can be of some help trying to assign a role to these cells.

The glomerular neuropile has a bipartite compartmental organization, demarcated by the presence or not of ON terminals (ON- non-ON zones; Chao et al., 1997; Kosaka et al., 1997; Kasowski et al., 1999); one-third (Hayar et al., 2004; Kiyokage et al., 2010) to half (Kosaka et al., 1997) of PG cells receive monosynaptic input from the olfactory nerve in the ON-zone (type 1 PG cells) – CR interneurons (and, interestingly, another important population of PG cells expressing a different CaBP, calbindin) are among the PG cells not directly in contact with the ORN, classified as type 2 PG cells,.

The majority of CR PG cells in mouse employ GABA as a neurotransmitter as shown by using GAD67 GFP knockin mice (Panzanelli et al., 2007) or immunohistochemistry (Kosaka and Kosaka, 2007), which means that in adult animals these neurones are inhibitory.

Another important feature of these neurones depends in their wiring scheme: since they are anaxonic (Kosaka and Kosaka, 2010), they can participate only to intraglomerular interconnectivity. Interglomerular connectivity is subserved by axonic PG cells, which probably contribute to lateral inhibition processing (Mori et al., 1999; Urban and Sakmann, 2002; Aungst et al., 2003; Arevian et al., 2008), a function that is considered to enhance the contrast between similar odors (Yokoi et al., 1995). The anaxonic PG cells, on the contrary, are likely involved in intraglomerular self-inhibition or self-modulation processes (Gire and Schoppa, 2009; Kosaka and Kosaka, 2011).

Finally, type 2 PG cells (mainly CR and calbindin subtypes), receive feed forward excitatory input from ORN via external tufted (ET) cells (Hayar et al., 2004; Batista-Brito et al., 2008; Gire and Schoppa, 2009; Kiyokage et al., 2010); it is estimated that a single ET cell activates about 5 PG cells in the same glomerulus (Murphy et al., 2005).

### Functional Implications

Due to their unusual complement of voltage-dependent conductances, CR PG cells show a phasic behavior, responding with a single action potential to a train of excitatory inputs sufficiently close to depolarize stably the membrane; this arises from the inactivation of the two main voltage-dependent conductances (A-type and Na), which rend the cells completely unexcitable. This means that in a train of close excitatory inputs only the first would produce an action potential – the cell would be unable to fire in response to subsequent inputs (**Figures 6** and **7**), and will enter in a kind of standby mode.

In principle, even if in an inactivated state, and therefore incapable to generate action potentials, the cell can still be depolarised by excitatory inputs, which could propagate electrotonically inducing the release of neurotransmitter in distant sites - there are very careful theoretical studies demonstrating that the structure of PG dendritic arborisation is compatible also with an electrotonic functioning of these cells (Rall et al., 1966; Rall and Shepherd, 1968), in some way similar to that of horizontal cells in the retina. However, the amount of neurotransmitter released, being governed by the increase of cytosolic Ca^2+^ concentration, would be effectively hampered by the presence of CR.

Under this aspect, the Ca-buffering capabilities of CR are interesting. This CaBP contains two pairs of high-affinity cooperative binding sites and one low affinity independent binding site (Schwaller et al., 1997; Stevens and Rogers, 1997; Faas et al., 2007). In a pair, initially the binding sites are in a state of low affinity and slow binding rate. When the first Ca^2+^ binding site is occupied, the other site changes to a state characterized by higher affinity and faster binding rate. The progressive increase in the Ca^2+^ association rate as a function of [Ca^2+^]_i_ results in an increased Ca^2+^ buffering capabilities by CR (Faas et al., 2007; Schwaller, 2009). This property would grant to CR PG cells the maximal increase in [Ca^2+^]_i_ (and therefore release of neurotransmitter) when the cells are excited from a resting state, much smaller when the cell is excited repetitively, and parallels well the excitability profile as determined by the complement of voltage-dependent conductances present.

Given these properties, we can make some inference about the role of these cells in the olfactory bulb. Since CR PG cells are inhibitory, and are positioned at the entry of the bulbar network, it can be suggested that their role could be improving the signal to noise ratio: random excitatory inputs, as those characterising meaningless signals (noise), would translate into an activation of these cells, and consequently into a inhibition of projection neurones. In this context, it should be reminded that CR PG cells are very small cells, with a capacity a little above 4 pF and a very high input resistance (about 1.9 GΩ), higher that the average PG cells (∼1 GΩ, (Puopolo and Belluzzi, 1998; Smith and Jahr, 2002), implying that they can be activated by weak excitatory inputs, a property of some importance if they role is that of cutting off weak signals.

The normal response of olfactory receptor neurons to odor detection, on the contrary, consists in high frequency trains of action potentials (Gesteland and Sigwart, 1977; Getchell and Shepherd, 1978; Sicard, 1986; Duchamp-Viret et al., 1999; Savigner et al., 2009; Tan et al., 2010; Martelli et al., 2013; Vaaga and Westbrook, 2016); a well-structured, persistent excitation of this kind, typically associated to a significant input (signal), would rapidly bring to an inactivated state the CR interneurons and buffering with maximal affinity and maximal rate the Ca^2+^ entering the cell, thereby silencing these inhibitory element and functionally excluding them from the network.

Ultimately, this would suggest the presence in the entry sector of the bulbar network of an element with limited firing abilities, “speaking” seldom, but capable to suppress the noise with well targeted interventions and leaving better organized signals to be further processed in the higher levels, in line with recent computational and experimental studies suggesting that the incoming olfactory information in the OB in its earlier stages is processed in a kind of parallel computational modality (Carey et al., 2015; Vaaga and Westbrook, 2016).

## Author Contributions

OB: Experimental design, data analysis, authorship of the manuscript. AP: Execution of the experiments, data analysis, and animal care. AFI: Execution of the experiments, data analysis, and animal care.

## Conflict of Interest Statement

The authors declare that the research was conducted in the absence of any commercial or financial relationships that could be construed as a potential conflict of interest.
