# Supplementary material for: Calretinin-Periglomerular Interneurons in Mice Olfactory Bulb: Cells of Few Words
Source: Front Cell Neurosci. 2016 Oct 7;10:231. doi: 10.3389/fncel.2016.00231 (PMC5054022; doi:10.3389/fncel.2016.00231)
Supplement: Supplementary file 1 [file Data_Sheet_1.DOCX]

# Supplementary material

## Cable properties

Input resistance: 1500 MΩ; membrane capacitance 4.07 pF

## Whole-cell conductances (nS)

*g*_Na_ = 12.7

*g*_A_ = 8.1

## Equilibrium potentials (mV)

*E*Na = +61.4

*E*K = −97.5

*E*leak = −60

## Sodium current − INa

### Steady-state activation:

*m_∞_(V)* = 1/(1+exp(−45.8−*V*)/8.1))

### Steady-state inactivation:

*h_∞_(V)* = 1/(1+exp(*V* +70/9.4))

### Activation time constant:

*τ_m_ (V)* = 0.07796 * exp (-V/21.752) + 0.00992

### Inactivation time constant:

*τ_h_ (V)* = 0.0679 * exp (-V/13.5) + 5.43

### Removal of inactivation time constant:

*τ_rh_ (V)* = 3853 * exp(-V/-17.58) + 5.11

## A-type potassium current − IA

### Steady-state activation:

*m_∞_(V)* = 1/(1+exp(−45.8−*V*)/8.1))

### Steady-state inactivation:

*h_∞_(V)* = 1/(1+exp(*V* +70/9.4))

### Activation time constant:

*τ_m_ (V)* = 0.07796 * exp (-V/21.752) + 0.00992

### Inactivation time constant:

*τ_h_ (V)* = 0.0679 * exp (-V/13.5) + 5.43

### Removal of inactivation time constant:

*τ_rh_ (V)* = 3853 * exp(-V/-17.58) + 5.11

## L-type calcium current - I_Ca_

The current has been modelled from the I/V relationship using the equation:

I_Ca_(V) = (-0.644 - 29.46 / (1 + 1.59432 * 2 * ((V - 2.43) / 49.24)^2^ + 0.4 * 2 * ((V - 2.43) / 49.24)^6^))

## Other specifications

The numerical simulations have been made using Quick Basic 64, v. 1000 (Microsoft, Redmont, WA). The time increments used for the reconstruction were 10 *μ*s, corresponding to a frequency of 100 kHz; several trials have been made at different time increments to exclude errors due to numerical approximation. No *post hoc* filtering has been applied.

The available data did not allow the reconstruction of the h-current, which however, from our experimental results, in normal saline exerts a significant effect only on the resting membrane potential and in times much longer than those used in the numerical simulations presented.
